# Our Contemporaries

**Published:** 1915-09

**Authors:** 


### OUR CONTEMPORARIES.

---

Child Betterment, July, comments as follows, after quoting from our editorial on Literal Fly Swatting.

''We endorse the position of the Buffalo Medical Journal so far as its opposition to enlisting children in the anti-fly crusade is concerned, but we beg leave to state that the editorial does not mention the chief objection to the proposed slaughter of the fly by children. WHY TEACH CHILDREN CRUELTY TO ANIMALS?''

Unfortunately, the advance of sanitary knowledge no longer allows us to teach humanity in the broad sense of sparing all forms of animal life. Let us omit reference to the utilization of animals for physiologic and pharmacologic purposes. The fact remains that children must be brought up to regard flies, musquitoes, various other insects and even such vermin as rats and mice, as dangerous enemies of the human race. That is why we did not allude to slaughter of the fly by children as chiefly objectionable because cruel. We don't know whether the ordinary forms of fly killing are cruel or not, in the customary sense of the term. We don't know just how far the sensation of insects is developed. We attempt no discrimination between ''warm blooded and cold blooded animals'' from the standpoint of humanity. The fact remains that the kindheartedness that begins ''Baby-bye, Here's a fly. Let us watch him—you and I'' is objectionable not only to the grammarian but to the sanitarian. Instead of teaching children that it is wrong needlessly to set foot on a worm, we must teach them deliberately to set foot on certain destructive caterpillars. We must warn them against playing the Good Samaritan to ''poor sick kitties'' picked up on the street. We have the harder problem of teaching children not to be cruel and nevertheless that certain forms of animal life are always potentially dangerous and all forms sometimes actually dangerous.

---

The Louisville Journal of Medicine and Surgery for August, says, sarcastically:

''It is nothing but natural that an astute lawyer who has cleared a murderer and turned him loose on the public should shake hands with and otherwise congratulate himself."

Considering the fact that the law of this and other enlightened countries guarantees to all persons charged with crime, a fair trial and the benefit of counsel, it occurs to us that a lawyer employed or assigned to defend a murderer may very reasonably take the ground that it is his duty to act sincerely for the interests of his client, and successfully if possible. In the case assumed, we imagine that the lawyer feels exactly the way that a physician does who has saved the life of a person whom he knows to be a detriment to society. In either case, for the professional man to fail to do his best or deliberately to act against the interests of his client or patient, because of his conception of the interests of society, shows ethical hypermetropia. The same logic would advocate betrayal of an army by a soldier who did not approve of his country's policy in a war.

---

The Journal of Inebriety has been reported discontinued on account of the death of the editor. Dr. T. D. Crothers of Hartford, who has been the sole editor-in-charge since the establishment of the journal in 1875 is very much alive. Owing to the sudden death of the publisher, a temporary suspension of publication was inevitable but plans have already been made to resume issue at an early date.

